# Certification and the Responsibilities Involved

**Published:** 1911-11-04

**Authors:** 


### MENTAL HOSPITAL MATTERS.

(Administrative and Practical Contributions and Photographs invited.)

### Certification and the Responsibilities Involved.

A scientific medical definition of insanity cannot yet be stated since it must be based on physiological and pathological knowledge which at present we do not possess. A legal definition would seem to be a necessity, but also obviously cannot be given since it implies the statement of a criterion of sanity. Up to the present we have had to get on without an absolute definition and have done so with surprisingly little squabbling. In ordinary cases where the matter in hand is care and treatment, the question whether a patient is insane is left by law to the opinion of one or more medical men and a magistrate, whose opinions have to be upheld, if the certificates are to continue in force, by the medical officer under whose care the patient is placed, and by the Commissioners in Lunacy. In special cases affecting property the question is argued in a court of law, where expert evidence is usually called on each side (usually, too, equally dogmatic), after which the judge decides the point. Certification is never desirable if it can possibly be avoided; but if proper treatment demands, it should not be delayed, as it gives legal power to enforce treatment irrespective of the wishes and desires of the patient. It is essential when, the patient being certifiably insane, money is paid for the maintenance or the case is sent to an institution. By "certifiably insane" is meant such a mental state that the physician can, on a personal examination, discover facts which he can put in writing to justify his opinion that the patient is a proper person to be taken charge of and detained under care and treatment as a person of unsound mind; and this implies that, in the physician's opinion, the placing under control is necessary to prevent the probability of something detrimental occurring to the patient or other people.

The medical man does not always recognise his responsibilities in certifying or refusing to certify his patient, but those responsibilities none the less exist to his patient, to other people, and to himself. If he certifies he is responsible with the petitioner to his patient for the stigma which certification will bring to the minds of lay people who know of it: if he refuse to certify he becomes responsible for preventing any irresponsible acts of his patient which may either prevent efficient treatment or lead to physical or moral harm. His responsibilities to the relatives of the patient are much the same as those to the patient, and to the rest of the world he makes himself responsible by not certifying for the patient not becoming a nuisance or danger. As to himself, the patient may subsequently bring an action at law against him for signing the certificate, and though there is a special clause in the Act to protect him if he acted *bona fide*, he may nevertheless find that defending the action has cost him both time and money.

